# Activation-induced cytidine deaminase targets SUV4-20-mediated histone H4K20 trimethylation to class-switch recombination sites

**DOI:** 10.1038/s41598-017-07380-9

**Published:** 2017-08-08

**Authors:** Virginia C. Rodríguez-Cortez, Paloma Martínez-Redondo, Francesc Català-Moll, Javier Rodríguez-Ubreva, Antonio Garcia-Gomez, Ganesh Poorani-Subramani, Laura Ciudad, Henar Hernando, Arantxa Pérez-García, Carlos Company, José M. Urquiza, Almudena R. Ramiro, Javier M. Di Noia, Alejandro Vaquero, Esteban Ballestar

**Affiliations:** 1 Chromatin and Disease Group, Cancer Epigenetics and Biology Programme (PEBC), Bellvitge Biomedical Research Institute (IDIBELL), 08908 L’Hospitalet de Llobregat, Barcelona, Spain; 2 Chromatin Biology Group, Cancer Epigenetics and Biology Programme (PEBC), Bellvitge Biomedical Research Institute (IDIBELL), 08908 L’Hospitalet de Llobregat, Barcelona, Spain; 3 Institut de Recherches Cliniques de Montréal, Division of Immunity and Viral Infections, Montréal, H2W 1R7 Québec Canada; 4 Division of Experimental Medicine, Faculty of Medicine, McGill University, Montreal, QC Canada; 5 B Cell Biology Lab, Centro Nacional de Investigaciones Cardiovasculares Carlos III (CNIC), Madrid, Spain; 6 Université de Montréal, Department of Medicine, Montreal H3T 1J4 Québec, Canada

## Abstract

Activation-induced cytidine deaminase (AID) triggers antibody diversification in B cells by catalysing deamination and subsequently mutating immunoglobulin (Ig) genes. Association of AID with RNA Pol II and occurrence of epigenetic changes during Ig gene diversification suggest participation of AID in epigenetic regulation. AID is mutated in hyper-IgM type 2 (HIGM2) syndrome. Here, we investigated the potential role of AID in the acquisition of epigenetic changes. We discovered that AID binding to the IgH locus promotes an increase in H4K20me3. In 293F cells, we demonstrate interaction between co-transfected AID and the three SUV4-20 histone H4K20 methyltransferases, and that SUV4-20H1.2, bound to the IgH switch (S) mu site, is replaced by SUV4-20H2 upon AID binding. Analysis of HIGM2 mutants shows that the AID truncated form W68X is impaired to interact with SUV4-20H1.2 and SUV4-20H2 and is unable to bind and target H4K20me3 to the Smu site. We finally show in mouse primary B cells undergoing class-switch recombination (CSR) that AID deficiency associates with decreased H4K20me3 levels at the Smu site. Our results provide a novel link between SUV4-20 enzymes and CSR and offer a new aspect of the interplay between AID and histone modifications in setting the epigenetic status of CSR sites.

## Introduction

Activation-induced cytidine deaminase (AID; gene symbol *AICDA*) is a key enzyme in B cell biology because it is needed to generate immunoglobulin (Ig) diversification by inducing class switch recombination (CSR) and somatic hypermutation (SHM)^1^. AID initiates SHM and CSR by deaminating cytosines to uracils. This leads to the generation of dU:dG mismatches that are differentially processed to generate double-strand breaks in Ig switch regions in CSR and point mutations in Ig variable regions during SHM. However, AID is also expressed in a variety of germ and somatic cells and there is evidence of additional roles. For instance, AID is homologous to the well-characterised RNA-editing enzyme APOBEC1, and its participation in RNA editing of viral genomes, perhaps in conjunction with an unknown cofactor, has been suggested^2^. AID can also deaminate numerous non-immunoglobulin genes, including *CD79A*, *MYC* and *PAX5* ^3–6^. The basis of the off-target deamination ability of AID are not fully understood but include its preference to interact with H3K27Ac-rich superenhancers^6^.

A variety of studies have demonstrated different connections between AID and epigenetic modifications at its binding sites. For instance, KAP1 and HP1 tether AID to H3K9me3 residues at the donor switch region^7^. Additionally, 14-3-3 adaptors proteins directly interact with AID and target it to the selected S regions, where 14-3-3 promotes recombination through the recognition of the open chromatin state in these S regions and its specific binding to phospho Ser10 and acetyl Lys9 histone H3^8^. Also, the presence of H3.3 in the nucleosomes of the Ig variable regions promotes the generation of AID-accessible single-stranded DNA that promotes Ig diversification^9^. AID-deficient mice have significantly lower levels of histone acetylation in S regions than observed in WT mice, implying a relationship between AID expression and histone modification enzymes^10^. It has also been proposed that AID’s catalytic activity, which mediates the conversion of cytosines to uracils, may participate in the removal of methylated cytosines as a two-step mechanism leading to active demethylation. In this context, AID would result in demethylating and activating critical pluripotency genes, OCT4 and NANOG, during reprogramming of somatic cells to pluripotent stem cells^11^. Despite these findings, the involvement of AID in active DNA demethylation seems to be restricted to specific scenarios^12^, and it remains controversial in the B cell context^13, 14^.

AID deficiency causes hyper-IgM type 2 (HIGM2) syndrome^15^, which is characterised by the absence of immunoglobulin CSR and SHM. The study of natural AID mutants associated with HIGM2 as well as engineered mutants has led to the characterisation of the active domains of the protein. AID, through its cytidine deaminase activity, induces targeted DNA lesions required for both CSR and SHM. Besides its cytidine deaminase activity, AID plays a further essential role in CSR, probably through the recruitment of CSR-specific cofactors by its C-terminus^16, 17^. A similar binding of SHM-specific cofactors to the N-terminal part is suggested by the functional characteristics of N-terminal AID artificial mutants. Finally, AID may act as a homo-, di-, or multimeric complex^18^. Together, these findings strongly suggest that AID acts on CSR not only as a cytidine deaminase enzyme, but also as a docking protein, recruiting specific cofactors to a multimeric complex. These interactions probably also include epigenetic enzymes and influence the epigenetic status of AID target sites, preparing the chromatin context for efficient CSR and SHM, and perhaps directly regulating its transcriptional status.

In the study reported here, we investigated the effects of AID on the epigenetic status of its binding sites, including the analysis of DNA methylation and several histone modifications. We observed a sharp increase in H4K20me3 at Sμ sites, which are also detected at the global level. In 293F cells, we demonstrated a physical interaction between co-transfected tagged AID and the SUV4-20H1 and SUV4-20H2 enzymes, involved in the dimethylation and trimethylation of H4K20. We also show that AID exchanges SUV4-20H1 and SUV4-20H2 at Sμ sites. The analysis of HIGM2 mutants of AID reveals that the truncated form W68X is impaired for efficient binding with SUV4-20H1 and SUV4-20H2 and H4K20me3 is no longer targeted to Sμ sites. Most importantly, we show that mouse primary B cells activated for CSR with LPS + IL4 undergo an increase in H4K20me3 that does not occur in AID-deficient mice. A similar association between AID and H4K20me3 levels at Sμ in B cells is obtained in CH12F3-2 cells, when they are depleted of AID and subsequently re-transfected with AID. Our results demonstrate for the first time the AID-dependent recruitment of SUV4-20 enzymes at Ig switch regions and suggest a direct interaction with AID, providing a novel link between these two enzymes that explains the effects on CSR of abrogating Suv4-20h enzymes in mice^19^.

## Results

### AID preferentially associates with heterochromatin

**Figure 1:**
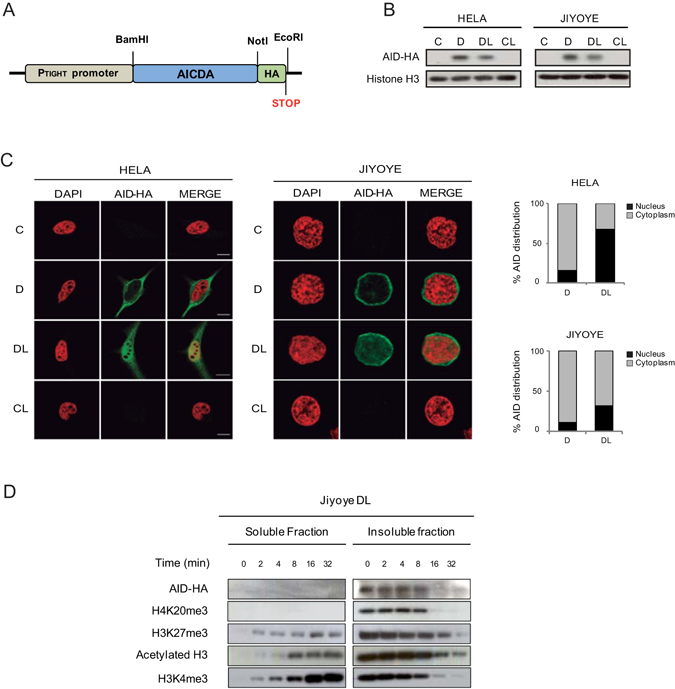
An inducible system for AID expression and accumulation in the nucleus. (**A**) Representative scheme of the HA-tagged AID retroviral construct. (**B**) AID expression is induced following doxycycline (D) treatment and retained in the nucleus upon additional treatment with leptomycin B (L), which specifically inhibits nuclear export. Anti-HA antibody was used to detect AID expression and histone H3 was used as a loading control (**C**) Representative confocal images of AID subcellular localisation in HeLa and Jiyoye cells after doxycycline and leptomycin B treatment. Nuclear DNA was counterstained with DAPI (red). A total of 25 cells from randomly selected fields were analysed in each experimental condition. The graphs in the right show the quantification of the cellular signal of AID within the cells. Light gray section of the bar indicates the average percentage of cytoplasmic AID signal. Black section of the bar indicates the average percentage of nuclear AID signal. (**D**) Association of AID with heterochromatin. Time course digestion of HA-tagged AID Jiyoye cells nuclei with DNase I, in which the supernatant contains the euchromatin fraction (as demonstrated by the appearance of H3Ac and H3K4me3) and the pellet contains the heterochromatin fraction (as shown by the progressive decrease of H4K20me3 and H3K27me3).

To investigate the ability of AID to target epigenetic modifications at its cognate sites in B cells, and how these changes may be impaired in HIGM2 individuals, we first generated an inducible retroviral system to express wild type AID (Fig. 1A). We selected two cell types, Jiyoye and HeLa, for our experiments. Jiyoye cells, which are Burkitt lymphoma-derived B cells, are a B cell model in which AID is not expressed. HeLa cells have been used for many biochemical studies of human AID and are ideal for testing the cell distribution by immunofluorescence. For the inducible system, we chose to infect Jiyoye and HeLa cells with the RetroX-Tet-ON advanced vector with geneticin resistance, followed by transduction with the pRetroX-Tight-Pur vector encoding AID tagged with HA at its C-t. AID was expressed following the addition of doxycycline (Fig. 1B). To ensure the accumulation of AID in the cell nucleus, nuclear export was inhibited by the addition of leptomycin B, as previously described^20^. As expected, we observed nuclear accumulation of AID-HA after leptomycin B treatment in Jiyoye and HeLa cells (Fig. 1C).

To determine whether the bulk of AID is associated with heterochromatin or euchromatin we performed an endonuclease digestion-based assay^21^ to compare the ability of AID to be released from chromatin with euchromatic (H3Ac and H3K4me3) and facultative heterochromatic (H3K27me3) and constitutive heterochromatic (H4K20me3) histone modifications. Using this assay strategy we observed that euchromatic histone modifications are released following digestion and are apparent in the supernatant after 2–4 min of digestion, whereas constitutive heterochromatic marks remain mostly in the insoluble fraction after long digestion. In this assay, AID displayed a similar behaviour to that seen in H4K20me3 (Fig. 1D), suggesting that it is associated with these modifications in the constitutive heterochromatin compartment.

### AID expression results in an increase in H4K20me3 at Switch repeats of the *IGH* locus

**Figure 2:**
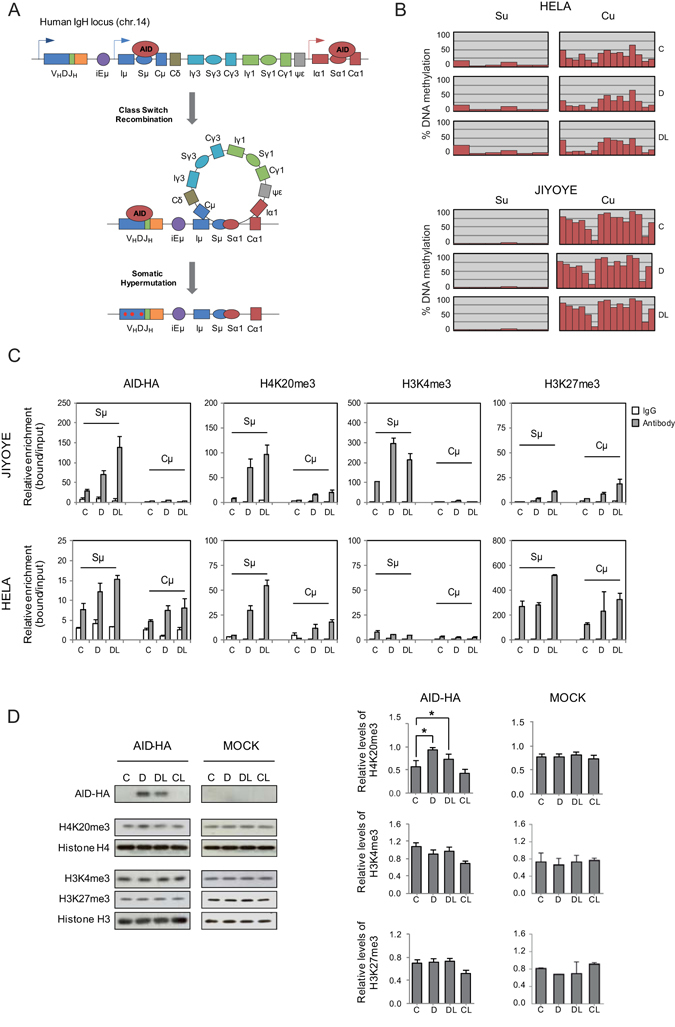
Effects of AID binding on the epigenetic status of the *IGH* locus. (**A**) Schematic representation of the *IGH* locus, and the participation of AID in class switch recombination and somatic hypermutation. Sμ and Cμ regions within this locus are *bona fide* binding and non-binding sites for AID and are used to test the effects of AID on epigenetic status. (**B**) Bisulphite pyrosequencing of the Sμ and Cμ regions in HeLa and Jiyoye cells transduced with the inducible retroviral system before (C) and after induction with doxycycline (D), and following inhibition of nuclear export with leptomycin B (DL). Each red bar shows the percentage of DNA methylation at a CpG site. 6 CpG sites were analysed at Sμ region, whereas 15 CpG sites were analysed at Cμ region (additional details in Supplementary Figure 1A). (**C**) AID association and selected histone modifications at the Cμ and Sμ regions using ChIP assays. AID was immunoprecipitated using anti-HA. ChIP assays also included H4K20me3, H3K27me3, H3Ac, H3K4me3 at the Cμ and Sμ regions. IgG was used as a negative control. For HeLa and Jiyoye cells we used control (C), doxycycline (D), and doxycycline +leptomycin B conditions (DL). Y-axis shows the relative enrichment of bound fraction with respect to input fraction. (**D**) Effects of AID on the global content of H4K20me3, H3K4me3 and H3K27me3 of Jiyoye cells as determined by western blot (left panel) and quantitation (right panel) of three independent experiments. Mock-infected cells were used as an additional negative control. Concomitantly with AID overexpression, the levels of H4K20me3 increased significantly (t- test p < 0.01) whereas H3K4me3 and H3K27me3 were unaffected.

CSR and SHM both depend on AID activity and its direct binding to specific sites at the Ig genes. To initiate productive CSR, AID-induced double-strand breaks (DSBs) must occur at the switch (S) repeat regions of the *IGH* locus that precede the participating constant (C) region gene segments (Fig. 2A and Supplementary Figure 1A). These are very well defined sequences that enable us to investigate the potential effect of AID on their epigenetic status. In this analysis, we first investigated the binding of AID to the Sμ segment in the two inducible cell models. ChIP assays revealed specific binding of AID to the Sμ following induction of expression and a further increase after inhibition of nuclear export. AID enrichment at the Sμ region could be observed in HeLa cells but was 8-10-fold higher in Jiyoye B cells, presumably because the *IGH* is much more strongly transcribed in the latter than the former. This binding did not occur at the Cμ sequence in Jiyoye cells and only to a limited extent in HeLa cells (Fig. 2C, left panel). To test the potential effect of AID on DNA methylation, we performed bisulphite pyrosequencing of specific CpG sites located within the Sμ and Cμ regions (Fig. 2A). We found no changes in the DNA methylation levels at either the Cμ region or the Sμ site, where AID binds, following doxycycline and leptomycin B treatment (Fig. 2B). In fact, when the DNA methylation status of these sites was compared at the genomic level between control and AID-expressing cells using methylation bead arrays we found no significant changes (Supplementary Figure 1B). Furthermore, the analysis of repetitive elements, such as Alu and LINE-1 repeats, also failed to find any changes (Supplementary Figure 1C and D), which rules out the existence of DNA demethylation events in association with AID binding, at least in this biological model.

We then performed ChIP assays with three different histone modifications, focusing on their association with the Sμ and Cμ regions. Specifically, we looked at H4K20me3, H3K27me3 and H3K4me3 (Fig. 2C). EZH2 and H3K27me3 modulate chromatin structure in B cell differentiation^22^. On the other hand, B-cell-specific conditional knockouts for the *Suv4-20h1* and *Suv4-20h2* genes, encoding for the enzymes that are mainly responsible for the balance between H4K20me3 and H4K20me2 results in defective in CSR, suggesting potential links to AID^19^.

We observed specific enrichment of H4K20me3 at Sμ but not at Cμ regions, both in Jiyoye B cells and HeLa cells following expression of AID and translocation to the nucleus (Fig. 2C) concomitant with the specific binding of AID to Sμ sequences previously demonstrated. Regarding H3K4me3, we also observed changes in Jiyoye B cells but not in HeLa cells. H3K27me3 was present in the Sμ and Cμ regions of HeLa cells but not in Jiyoye cells.

To determine whether changes in H4K20me3 also occurred at the global level we performed western blot assays with Jiyoye cells before and after induction with doxycycline. No changes at the global level were observed for H3K27me3 and H3K4me3 (Fig. 2D). We found a significant increase in H4K20me3 following AID induction, reinforcing the notion of a direct role of AID in producing an increase in this modification at its binding sites (Fig. 2D). Because of this global effect together with the potential role of Suv4-20 family members in CSR, we decided to explore a mechanistic link between AID and the methyltransferases responsible for H4K20 methylation.

### Transfected AID interacts with Suv4-20 family proteins and recruits them to chromatin in 293F cells

**Figure 3:**
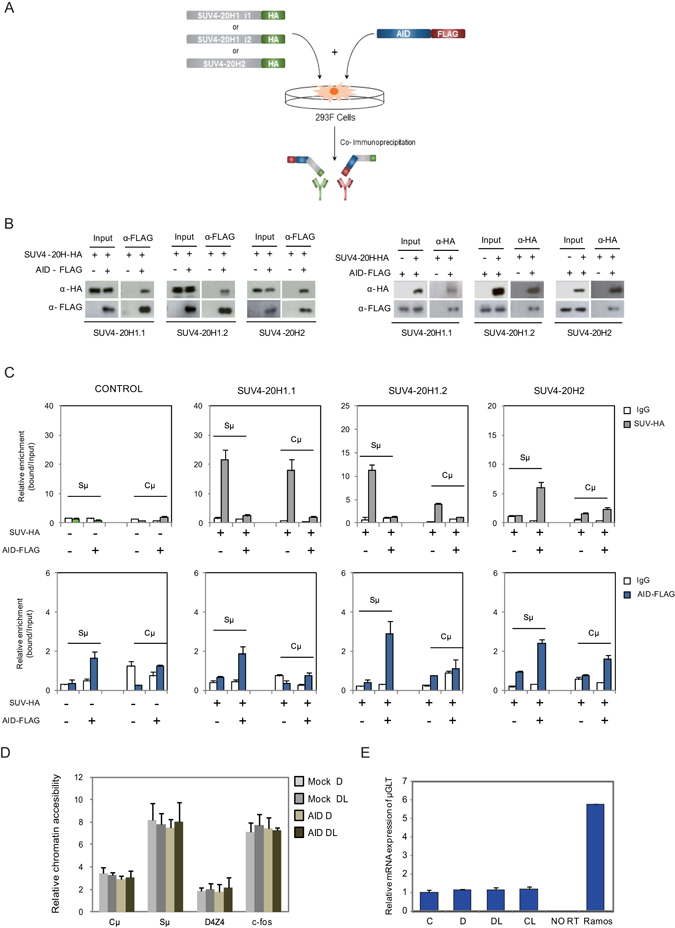
AID interacts with SUV4-20H enzymes and recruits them to IgH Sμ regions (**A**) Scheme depicting immunoprecipitation experiments of 293F cells co-transfected with Flag-tagged AID and each of the HA-tagged SUV4-20H enzymes: SUV4-20H1.1, SUV4-20H1.2 and SUV4-20H2. AID was also transfected on its own. Material immunoprecipitated with anti-HA was then blotted and visualised with anti-FLAG to determine its potential interaction with AID. The reciprocal experiment was also performed, using anti-FLAG to immunoprecipitate AID and anti-HA was used to test the interaction with each of the SUV4-20H enzymes. (**B**) Western blot assays showing the results of the above experiment (**C**) ChIP assays showing the recruitment of AID and SUV4-20H enzymes at the Cμ and Sμ regions. 293F cells were co-transfected or not with AID-FLAG construct plus the different SUV4-20H-HA enzymes constructs. After 24 hours, cells were fixed, lysed and chromatin sonicated to perform ChIP assays with control IgG, anti-FLAG antibody to immunoprecipitate AID or anti-HA antibody to pull down SUV4-20H enzymes. The upper section shows the association of SUV4-20H enzymes with the Sμ and Cμ regions. Light gray bars indicate the relative enrichment of SUV4-20H enzymes (HA antibody) with respect to input fraction. White bars represent negative control of enrichment (IgG). Lower section of the figure shows the association of AID with the Sμ and Cμ regions. Blue bars indicate the relative enrichment of AID (FLAG antibody) with respect to input fraction. White bars represent negative control of enrichment (IgG). (**D**) DNA accessibility assay. Amplification by quantitative PCR of Sμ, Cμ, D4Z4 and c-fos regions after DNAse I digestion. Accessibility is represented as the ratio among the final reaction point (30 minutes) and the initial reaction point (1 minute). It was possible to detect variations in DNA accessibility levels as demonstrated by the differences detected among D4Z4 and c-fos regions, but it was not detected variations in DNA accessibility after AID overexpression and its nuclear accumulation. This experiment was carried out with Jiyoye cells transduced with empty vector (Mock) or with AID encoding vector. The DNA accessibility was assessed in these cells after treatment with doxycycline (**D**) or doxycycline + LMB (DL). (**E**) μGLT expression after AID overexpression and nuclear accumulation. The μGLT expression was normalized to RPL38 expression. Ramos cells were used as a positive control of μGLT expression, whereas Jiyoye sample processed without retrotranscriptase was used as a control of genomic DNA contamination.

Changes in H4K20me3 following binding of AID may suggest an association between AID and the recruitment of histone methyltransferases of the Suv4-20 family. As aforementioned, there are two genes in this family, *SUV420H1* and *SUV420H2*, the first of which produces two isoforms, SUV4-20H1.1 and SUV4-20H1.2, through alternative splicing (Fig. 3A). SUV4-20H1.1 and SUV4-20H1.2 are mainly H4K20 dimethyltransferases (monomethylation is achieved through PR-SET7) and SUV4-20H2 is involved in the transition from dimethyl to trimethyl H4K20 and is a major constituent of heterochromatin^23^. We performed co-immunoprecipitation experiments to explore the potential physical interactions between these enzymes and AID (Fig. 3A).

In these experiments, we used an alternative cell system 293F, which allows the efficient co-transfection of tagged SUV4-20H proteins and AID, and leads to the co-expression of the C-t FLAG-tagged AID along with an HA-tagged version of each of the SUV4-20 enzymes. The use of this alternative model allows us to test each SUV4-20 enzyme individually because there are no specific antibodies for the three of them. These experiments revealed that AID interacts with the three SUV4-20H proteins, as demonstrated by immunoprecipitation of Flag-tagged AID and the reciprocal experiment immunoprecipitating with HA-tagged SUV4-20H proteins (Fig. 3B).

We then investigated the association of the SUV4-20H enzymes with the IgH Sμ and Cμ sites in relation to AID expression in these cells. ChIP experiments revealed that SUV4-20H1.1 is associated with the *IGH* Sμ region only in the absence of AID (Fig. 3C). Furthermore, co-transfection with AID impairs its association with the Sμ and Cμ sites (Fig. 3C). In the case of SUV4-20H1.2, we observed a similar behaviour, although this enzyme only associates with the Sμ site (Fig. 3C). Most importantly, we found that SUV4-20H2 only associates with the Sμ site in the presence of AID, suggesting a role for AID in recruiting this enzyme to the chromatin in these regions.

Since H4K20me3 is a mark of heterochromatin, we decided to investigate the potential impact of AID on accessibility at the IgH locus using DNaseI digestion followed by amplification by quantitative PCR with primers for the Cμ and Sμ sequences. We cut the DNase-resistant fraction (size range ≥5000 bp) at different times and amplified the aforementioned sequences. This strategy allowed us to monitor the dynamics of digestion and to estimate the accessibility to DNase. Analysis of these sequences in the Jiyoye cells inducible for AID expression revealed that the Cμ site and non-satellite subtelomeric repeats (D4Z4) are less accessible to DNase than the Sμ site or an active gene like c-Fos, which was used as a positive control. However, no differences were observed with respect to the expression or nuclear presence of AID (Fig. 3D).

We also checked whether AID association and binding to the Sμ IgH affects the levels of the transcription produced by the TSS related to this site, assessed by quantitative RT-PCR. No differences were seen with respect to the expression of AID (Fig. 3E).

### HIGM2 AID truncated form W68X is impaired for efficient binding to SUV4-20H1.2 and SUV4-20H2

**Figure 4:**
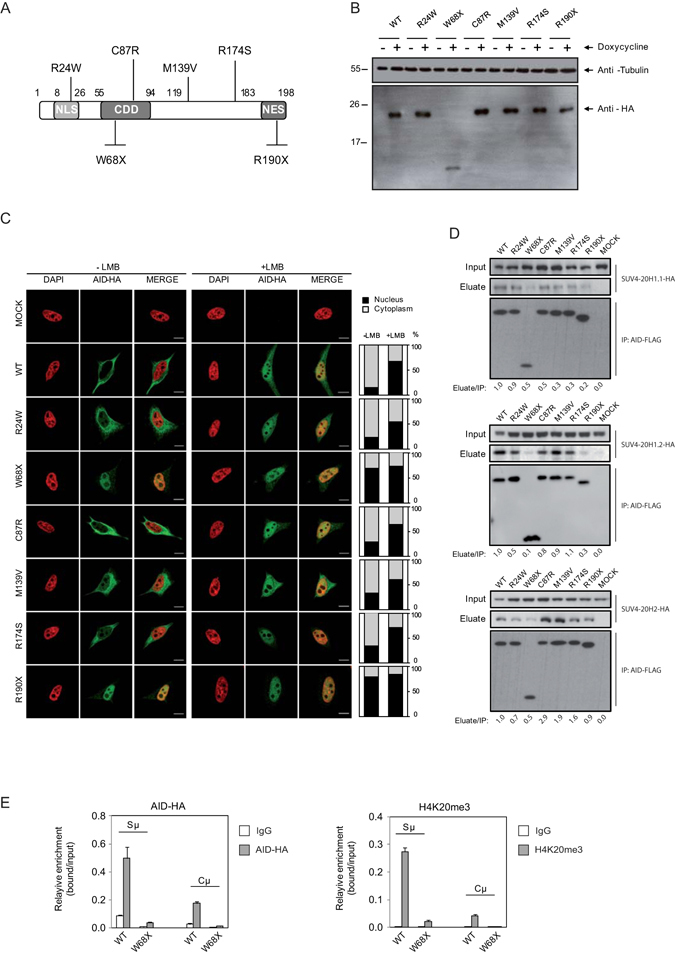
Effects of HIGM2 mutant forms of AID on cell distribution and interaction with SUV4-20H enzymes. (**A**) Primary structure of AID. The upper part of the diagram indicates the four missense mutations related to HIGM2used in our study. The lower part indicates the two selected nonsense HIGM2 mutations. NLS, nuclear-localisation signal; CDD, cytidine deaminase domain; NES, nuclear-export sequence. (**B**) Western blot image showing the inducible expression of AID WT and the various HIGM2 mutants, before and after treatment with doxycycline 500 ng/ml for 48 hours. (**C**) Representative confocal images showing the subcellular localisation of C-terminally hemagglutinin (HA)-tagged human AID in inducible HeLa cells. A total of 20 cells from randomly selected fields were analysed in each experimental condition. The graphs next to the confocal images, show the quantification of the cellular signal of AID within the cells. Light gray section of the bar indicates the average percentage of citoplasmic AID signal. Black section of the bar indicates the average percentage of nuclear AID signal. When nuclear export was inhibited with 50 ng/ml leptomycin B (LMB) for 2 hours, most of the AID translocates from the cytoplasm to the nucleus. Protein products of missense HIGM2 mutations showed a similar response to AID WT after LMB, while truncated forms of AID lacking NES were constitutively nuclear. Scale bar: 10 μm. (**D**) Co-immunoprecipitation of AID WT and HIGM2 mutants with the three SUV4-20H enzymes. All the HIGM2 mutants were also able to interact with the three SUV4-20H enzymes. (**E**) ChIP assays showing the recruitment of wild type AID (WT) and W68X AID mutant (W68X) and H4K20me3 enrichment at the Cμ and Sμ regions in Jiyoye cells after doxycycline and LMB treatment for 24 hours. AID was immunoprecipitated using anti-HA and ChIP assays also included anti-H4K20me3 antibody and IgG as a negative control. Y-axis shows the relative enrichment of bound fraction with respect to input fraction.

Our experiments demonstrated that AID and SUV4-20H enzymes can interact, and the effects of such interaction at Sμ sites. We wondered whether these interactions are impaired in the context of HIGM2 mutations. These experiments not only help us understand the functional implications of these mutations but also can help tease apart the roles of different domains in establishing these interactions. To this end, we generated a version of the AID-inducible system in HeLa and Jiyoye cells for several HIGM2 forms, including AID R24W, W68X, C87R, M139V, R174S and R190X (Fig. 4A). R24W AID is mutated in the nuclear localisation signal (NLS), W68X and C87R are, respectively, a nonsense and a missense mutation at the catalytic domain of AID. W68X produces a severely truncated protein (Fig. 4B). M139V and R174S are both missense mutations outside any known functional domain of AID. Finally, we also tested R190X AID, a nonsense mutant form that generates a shorter protein without part of the nuclear export signal (Fig. 4B). Interestingly, this region has recently been found to participate in recruiting DNA damage response factors^17^.

To test our mutants, we first investigated their cellular localization by immunofluorescence and confocal microscopy using a rabbit monoclonal anti-HA antibody. As mentioned above, wild-type AID is expressed following treatment with doxycycline and accumulates in the nucleus after leptomycin B treatment in HeLa and Jiyoye cells (Fig. 4C and Supplementary Figure 2). The R24W, C87R, M139V AID mutants had a similar behaviour to wild type AID, although the two truncated forms of AID, W68X and R190X, which contain the nuclear import signal but lack the nuclear export and cytoplasmic retention signals, accumulated in the nucleus constitutively^20, 24^ (Fig. 4C and Supplementary Figure 2).

We then tested the ability of these mutant forms of AID to interact with SUV4-20H enzymes. Co-immunoprecipitation experiments with the anti-FLAG antibody in cells co-transfected with HA-tagged AID and each of the three SUV4-20H enzymes showed that wild type AID and all the mutant forms can interact with SUV4-20H1.1, SUV4-20H1.2 and SUV4-20H2. The AID-truncated form, W68X, which lacks two thirds of the protein, was significantly impaired for binding for all SUV4-20 enzymes (Fig. 4D). Other mutant forms appeared to have various degrees of interaction, compared to wild type AID, however we were unable to have conclusive results for these other mutants due to interexperimental variation. These results indicated that the W68X form of AID might be unable to recruit SUV4-20H2 to Sμ sites. We therefore tested the ability of W68X to target H4K20me3 at the Sμ site in comparison with wild type AID. In ChIP experiments, we observed that, after induction with doxycyclin and LMB, the W68X AID form is unable to bind to the Sμ site of the IGH locus, in contrast with wild type AID. Consistently, no enrichment for H4K20me3 was observed for W68X AID (Fig. 4E), reinforcing the notion that H4K20me3 enrichment is associated with AID targeting of SUV4-20H2 to the Sμ site.

### AID targets H4K20me3 at Sμ in mouse primary B cells undergoing CSR

**Figure 5:**
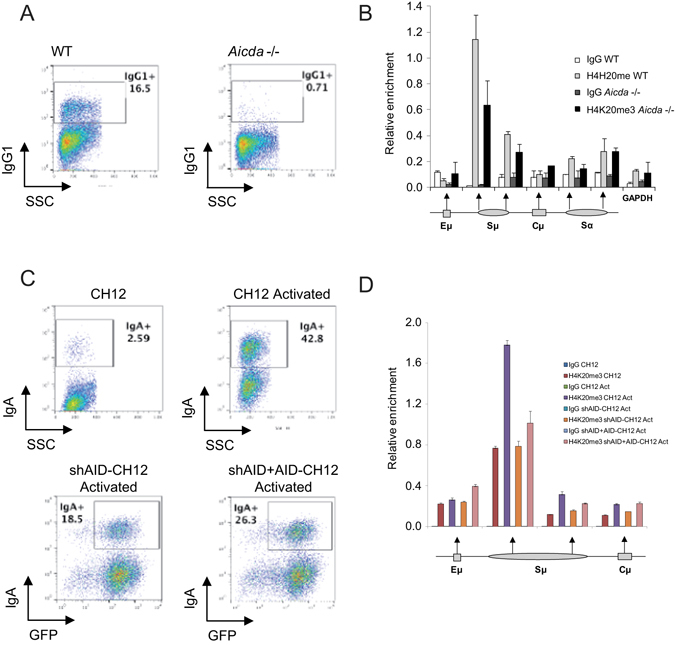
Role of AID in H4K20me3 deposition at *IgH* locus in switching primary B cells. (**A**) Primary B cells were isolated from wild type (WT) or *Aicda*−/− mice and then activated with LPS (5 μg/ml) and IL4 (30 ng/ml) for 3 days. After that, cells were stained to check class-switch recombination to IgG1. (**B**) Cells were also fixed, lysed and chromatin sonicated to perform ChIP assays with control IgG or anti-H4K20me3 antibodies. H4K20me3 deposition at IgH locus was analyzed using primers for specific regions where AID binding has been previously reported. Y-axis shows the relative enrichment of bound fraction with respect to input fraction. (**C**) Mouse CH12F3-2 cells were activated with 1ng/ml recombinant human TGF-β1, 10 ng/ml recombinant murine IL-4, and 1 µg/ml functional grade purified anti–mouse CD40 for switching to IgA. Additionally, CH12F3-2 cells stably expressing shRNA against mouse AID were complemented with human AID by retroviral transduction and then activated in the same conditions. CSR was analyzed 72hrs after activation by flow cytometry, after staining cells with Anti-Mouse IgA-PE and propidium iodide, for excluding dead cells. (**D**) For ChIP, CH12F3-2 cells were also fixed, lysed and chromatin sonicated to perform ChIP assays with control IgG or anti-H4K20me3 antibodies. H4K20me3 enrichment at IgH locus was analyzed using primers for specific regions where AID binding has been previously reported. Y-axis shows the relative enrichment of bound fraction with respect to input fraction.

Finally, to prove the relevance of our findings in primary B cells that are able to undergo CSR, we isolated resting B cells from both wild type C57BL6/J and *Aicda* knockout (*Aicda*−/−) mice and compared the ability of these cells to acquire H4K20me3 at the IgH locus (including Sμ sites) upon LPS- and IL-4-induced activation. LPS and IL-4 induced CSR to IgG1 in around 16.5% cells towards (measured as IgG1+ cells) in wild type B cells, whereas only a background 0.71% B cells were IgG1+ in *Aicda*−/− mice (Fig. 5A). ChIP analysis of those cells showed that H4K20me3 is highly enriched in LPS/IL-4-activated WT B cells at Sμ upstream site of LPS/IL-4-activated wild type B cells, where binding of AID has been previously described^25^ and such enrichment was significantly reduced in LPS/IL4-activated *Aicda*−/− B cells (Fig. 5B), demonstrating that AID presence or activity is associated to the occurrence of this modification.

We also performed ChIP assays using the mouse lymphoma cell line CH12F3-2, capable of efficient *in vitro* cytokine-induced AID expression and CSR to IgA through the same molecular mechanisms used by primary lymphocytes. We compared the H4K20me3 levels at the Sμ site in CH12F3-2 (CH12) cells before and after activation, in stable AID-knockdowns of CH12F3-2 cells (shAID-CH12) and shAID-CH12 re-infected with AID (shAID + AID-CH12). Knockdown and reintroduction of AID resulted respectively in reduction and increase CSR to IgA (Fig. 5C). In ChIP assays, we observed an increase of H4K20me3 at the Sμ site, which was respectively decreased and increased in parallel with the knockdown and reintroduction of AID (Fig. 5D), supporting further the role of AID in targeting H4K20me3 changes.

## Discussion

Our results demonstrate a novel relationship between AID and SUV4-20H enzymes that changes the H4K20 methylation status at the Sμ sites of the IgH locus. This result provides a novel link between AID and SUV4-20H histone methyltransferases that explains previous observations on the effects on CSR of knocking out Suv4-20h enzymes in mice^19^. The existence of such changes in the methylation status of H4K20 at these sites suggests a role for this process that is relevant to CSR and perhaps to SHM. Our results of AID HIGM2 mutants suggests that that the physical presence of AID contributes to recruit SUV4-20H2 to the Sμ sites. Changes in the H4K20me3 at the *IGH* locus are relevant in primary B cells undergoing CSR, and the loss of AID is accompanied by reduction in H4K20me3 levels at Sμ sites. Of note, the latter results also show that there is also AID-independent deposition of this mark at the S region. Our model system also revealed the lack of effects on DNA methylation, suggesting that the contribution of the cytidine deaminase activity of AID in DNA methylation, if any, is restricted to specific situations and is not simply associated with its high level of expression, but perhaps is more closely related to differentiation processes.

One of the most significant findings of our study is the identification of the link between AID and H4K20 methylation changes. H4K20 methylation is a chromatin modification that has been linked with diverse epigenetic functions. Enzymes responsible for H4K20 methylation are members of the SUV4-20H family, in which SUV4-20H1.1 and SUV4-20H1.2 are associated with H4K20 mono- and dimethylation and SUV4-20H2 catalyses H4K20 trimethylation. The generation of conditional null alleles for the two Suv4-20h histone methyltransferase genes in the mouse has been used to investigate the roles of H4K20 methylation states. Interestingly, Suv4-20h-double-null (dn) mice, which lose nearly all H4K20me3 and H4K20me2 states, are defective in immunoglobulin CSR, and this deficiency impairs the stem cell pool of lymphoid progenitors^19^. These results are consistent with our own finding of the direct participation of AID in the recruitment of SUV4-20H enzymes to CSR sites.

Several lines of evidence point to the epigenetic mechanisms as being key regulators of the events associated with antibody affinity maturation. SHM and CSR involve important alterations in DNA structure, and epigenetic mechanisms have been shown to be crucial not only for the establishment of the proper DNA conformation during these processes, but also for the recruitment of the necessary machinery.

The targeting of AID activity to specific DNA regions has been one of the most intensively studied aspects of B cell activation, yet it is still far from fully understood. Most of the research efforts related to epigenetic mechanisms and antibody affinity maturation have focused on describing the epigenetic landscapes that contribute to AID targeting and binding of CSR and SHM and, accordingly, AID recruitment has been associated with specific histone marks and specific DNA methylation patterns.

Our first observation was that, following DNase I chromatin digestion, AID presented a similar release pattern to that exhibited by the H4K20me3 heterochromatic mark. It is well established that AID is targeted to actively transcribed open chromatin regions, and its localisation is associated with active histone marks^26^, so we expected to see a coincidence of the release pattern of AID with the active histone marks represented by H3K4me3 and H3Ac in our experiment. Unexpectedly, AID was absent from the soluble fraction of DNase I digestion and mirrored the pattern of release of H4K20me3 in the insoluble fraction of the digestion. This fraction is not only associated with constitutive heterochromatin, but also contains regions of chromatin associated with large multimeric (transcriptional and DNA-repair) protein complexes^27^, which we presume are the regions that explain the partitioning of AID in this fraction. To date, many histone modifications have been shown to modulate the recruitment of AID to the Ig and AID expression correlates with changes in the acetylation of histones at the S regions. However, this is the first description of AID directly recruiting a histone modifying enzyme that affects the epigenetic status of the Ig locus.

Our results demonstrate a direct interaction between AID and SUV4-20H enzymes. This has been demonstrated in 293F cells, given the limited co-transfection levels of AID and SUV4-20H in B cells. Analysis of HIGM2 AID mutant forms reveals that these interactions can be lost in severe truncated forms like W68X. While in some cases, interactions with AID have been demonstrated to take place at its N-t, including the histone chaperone Spt6^28^, most interactions occur at the AID C-t, including those with the 14-3-3 adaptor proteins^8^, the translation elongation factor 1 alpha (eEF1A)^29^, or the germinal center-associated nuclear protein^30^. In the case of the W68X AID form, the loss of interaction with SUV4-20H1.2 and SUV4-20H2 suggests that the amino acid motifs after residue 68 of AID are necessary to interact with these enzymes and target H4K20me3 at the switching regions. There are additional evidences on the direct role of AID to target histone modifications to S regions, also in the context of B cell activation. For instance, AID-deficient B cells showed reduced levels of acetylation in H3 and H4 histones in S regions. Thus, the increase of H3Ac and H4Ac levels observed during B cell activation depends on AID expression^10^.

The increase in the H4K20me3 levels induced by AID overexpression in our cell system did not result in a change in the accessibility of S regions or alterations in the transcription of the μGLT. Therefore, there is no clear evidence of the role of this change during CSR. Recently, it has been proposed that hMOF-mediated H4K16Ac and SUV4-20H2-mediated H4K20me3 play opposing roles in the regulation of Pol II pausing. H4K16Ac promotes the release of Pol II from pausing, whereas Pol II remains paused in the presence of H4K20me3^31^. It is well known that the stalling of Pol II is a crucial step for AID targeting. Stalled Pol II not only acts as a docking protein for AID recruitment through Spt5 interaction^32^, but also facilitates the recruitment of histone modifiers to produce the accessibility required for AID activity. On the basis of these findings, we suggest that AID reinforces the stalling of Pol II by recruiting SUV4-20H2 and the increase of H4K20me3 levels in the Sμ region, thereby generating a positive feedback loop that reinforces its recruitment to mediate CSR. Further research is required to confirm this hypothesis.

Our results provide evidence of a novel AID role beyond its catalytic activity during antibody affinity maturation. The discovery of AID partners and the function of the variety of interactions established by AID is a crucial step towards fully understanding the additional roles of this enzyme during CSR and SHM, and also in B cell differentiation and maturation.

## Methods

### Human Cells

The human cell lines used in this study were grown in a humidified incubator at 37 °C and 5% CO_2_ and were maintained in log phase growth by changing the culture medium every 48–72 hours. The Burkitt lymphoma-derived cell line Jiyoye was cultured in RPMI medium supplemented with 5% FBS (v/v) and antibiotic/antimycotic solution; adherent HeLa and 293F cell lines were grown in DMEM medium with 5% FBS (v/v) and antibiotic/antimycotic solution.

### Mouse B cells and CSR

Resting B cells were purified from spleens of C57BL6/J mice or *Aicda*−/− mice (obtained from T. Honjo, Kyoto University, Sakyo-ku, Kyoto, Japan) as described previously^24^. Briefly, leukocytes were purified by densitiy cushion using Lympholyte-M (Cedarlane Labs), stained anti-CD43 microbeads (Miltenyi Biotech) and depleted of CD43+ cells using autoMACS Pro Separator (Miltenyi Biotech). The purified B-cells were plated at 0.5 × 10^6^ cells/ml and activated with 5 µg/ml LPS (Sigma) and 30 ng/ml recombinant murine IL-4 (Preprotech) for switching to IgG1. For ChIP, primary cells were cross-linked 48 h after activation as described below. In primary B cells. CSR was analyzed 72 h after activation by flow cytometry. Cells were incubated with FcR Blocking Reagent for mouse (Miltenyi Biotech); then stained with anti–IgG1-biotin (BD), followed by APC-conjugated anti-biotin antibody (Miltenyi Biotech). Dead cells were excluded from this analysis by propidium iodide staining.

Mouse CH12F3-2 cells were plated at 0.1 × 10^6^ cells/ml and activated with 1ng/ml recombinant human TGF-β1 (R&D systems), 10ng/ml recombinant murine IL-4 (Preprotech), and 1 µg/ml functional grade purified anti–mouse CD40 (BD) for switching to IgA. Additionally, CH12F3-2 cells stably expressing shRNA against mouse AID (gift from R. Verdun, University of Miami, Florida, Miami, USA) were complemented with human AID by retroviral transduction using the supernatant of HEK293 cells co-transfected with vectors encoding VSV-G and GAG-Pol and either the empty pMX-IRES-GFP vector or pMX-kozak-AID-IRES-GFP vector encoding AID. For ChIP, CH12F3-2 cells were cross-linked 24hrs after activation as described below. In CH12F3-2 cells, CSR was analyzed 72hrs after activation by flow cytometry, after staining cells with Anti-Mouse IgA-PE (Southern Biotech) and propidium iodide, for excluding dead cells.

### DNA constructs and the system for inducible AID expression

A C-terminally haemagglutinin (HA)-tagged AID was generated by PCR amplification with the forward primer 5′-ATGGATCCAGACACTCTGGACACCACTATG-3′ and reverse primer 5′-TAGAATTCCTAAGCGTAATCTGGAACATCGTA-3′. The forward and reverse primers respectively introduced a BamHI and an EcoRI site. The amplified fragment corresponding to AID WT was then cloned into BamHI/EcoRI-digested pRetroX-Tight-Pur vector. The AID mutants were generated by the Quickchange method (Stratagene) using the primers listed in Supplementary Table 1. To generate the inducible expression of AID we used the Retro-X^TM^ Tet-ON^®^ Advanced Inducible Expression System (Clontech). Firstly, Jiyoye and HeLa cell lines were transduced with the RetroX-Tet-ON advanced vector and geneticin selected at 1 mg/ml (Life Technologies). Secondly, cells were transduced with the pRetroX-Tight-Pur vector encoding AID and selected with puromycin (Sigma-Aldrich) at 0.3 µg/ml for Jiyoye cells and 3 µg/ml for HeLa cells. AID expression was induced by the addition of doxycycline (Clontech) at 500 ng/ml to the culture media for 24 hours for Jiyoye cells and 48 hours for HeLa cells. Nuclear export was inhibited by the addition of leptomycin B (LMB) (LC labs) for 2 hours at 10 ng/ml for Jiyoye cells and 50ng/ml for HeLa cells as previously described^20^. The experimental procedures with the inducible system for AID expression were carried out under four conditions: (1) Control (C): Cell lines without AID expression; (2) Doxycycline (D): Cell lines after doxycycline treatment to induce AID expression; (3) Doxycycline + LMB (DL): Doxycycline and LMB treatment to induce AID expression and its nuclear accumulation; and (4) Control + LMB (CL): Cell lines with LMB.

### Western blot

Proteins were separated on 15% or 8% SDS-PAGE gels and blotted onto a polyvinylidene difluoride membrane of 0.22-µm or 0.45-µm pore size (Immobilon PSQ, Millipore) according to the size of the protein analysed. The membrane was blocked in 5% milk TBS-T (Tris-buffered saline with 0.1% Tween-20) and immunoprobed with the antibodies listed in Supplementary Table 2. The secondary antibodies used were goat anti-rabbit-conjugated to horseradish peroxidase (HRP) (1:10,000–1:30,000) (Amersham) and sheep anti-mouse-HRP (1:5000). Experiments were performed in triplicate. Bands were quantitated by direct scanning of the western blot films with an HP Scanjet 4890 and processed with ImageJ software. Where indicated, a student t-test was applied to compare the levels of the analysed proteins (statistical significance *p < 0.01).

### Immunofluorescence and confocal microscopy

Immunofluorescence experiments were carried out as previously described^20^ with slight modifications. Briefly, the transduced the Jiyoye cell line was plated in a 24-well plate and allowed to attach to poly-lysine-coated coverslips for 15 min at 37 °C in PBS. HeLa cells were plated in a 6-well plate and allowed to attach to coverslips 24 hours before AID expression induction. After this time the cells were fixed with 4% paraformaldehyde, permeabilised in 0.5% (v/v) Triton X-100 and blocked with 1 mg/ml BSA + 5% goat normal serum in PBS. Both cells types were stained with anti-HA (1:200; Sigma-Aldrich) followed by anti-rabbit AlexaFluor 488 (1:1000; Life Technologies). Stained preparations were mounted in Mowiol-DAPI mounting medium and confocal optical sections were obtained using a Leica TCS SP5 Spectral confocal microscope (Leica Microsystems). Images were acquired with Leica Application Suite Advanced Fluorescence (LAS AF) software and processed with ImageJ software. Each immunofluorescence experiment was performed independently at least two times.

### DNAse I digestion-based assay

With this assay we followed the release of AID and various histone marks after DNase I digestion. The digestion was carried out in nuclei isolated from the Jiyoye cell line after treatment with doxycycline and LMB to induce AID overexpression and nuclear accumulation, respectively. Cell cytoplasms were disrupted with RSB buffer (10 mM Tris-HCl pH 7.5, 10 mM NaCl, 3 mM MgCl_2_). The isolated nuclei were then washed and resuspended in DNase I buffer before adding DNase I to initiate DNA digestion at 37 °C. We monitored the time course of DNase I-induced release of AID and histone proteins after 2, 4, 8, 16 and 32 minutes of digestion. The reaction was stopped by adding EDTA 0.5 mM. These procedures were carried out in the presence of Roche Complete Protease Cocktail Inhibitor.

Sensitivity to nuclease digestion is determined by chromatin structure. After DNase I digestion it is possible to isolate a highly nuclease-accessible chromatin fraction and a DNase-resistant fraction. The highly accessible chromatin fraction (soluble fraction) is mainly associated with active genes and therefore with activation-associated histone marks, whereas the DNase-resistant fraction (insoluble fraction) is mainly associated with constitutive heterochromatin and regions of chromatin associated with large multimeric (transcriptional and DNA-repair) protein complexes^27^.

From the samples taken at each digestion time we obtained soluble and insoluble protein fractions and subjected them to western blot analysis to determine the pattern of appearance or disappearance of AID and the specific histone marks H3K20me3 (heterochromatin mark), H3K27me3 (facultative heterochromatin mark), and H3K4me3 and acetylated H3 (euchromatin marks).

### Bisulfite pyrosequencing of Sμ and Cμ at the *IGH* locus

The DNA methylation status of the Sμ and Cμ regions, was determined by pyrosequencing fragments of 331 bp and 215 bp respectively (Supplementary Figure 1). Biotinylated amplicons for each gene region were generated by PCR using the HotStart Taq DNA polymerase PCR kit (Qiagen). Specific primers were designed using the PyroMark Assay Design Software (QIAGEN- version 2.0.01.15). Pyrosequencing reactions and quantification of DNA methylation were performed with Pyromark™ Q24 system (Qiagen). Results from bisulfite pyrosequencing are presented as a percentage of DNA methylation. Primers are listed in Supplementary Table 1.

### Expression of μ Germline transcripts

To analyze the expression of μ Germline transcripts (μGLT) of the *IGH* locus, we isolated total RNA with Trizol® Reagent (Life Technologies). Then, mRNA was retrotranscribed with the Transcriptor First Strand cDNA Synthesis Kit (Roche). cDNA synthesis reaction was carried out by using anchored oligo(dT)18 primer, especially designed to enrich the retrotranscription of mRNAs. We analyzed the expression of mature μGLT by amplifying a 106 bp region within the non-coding Iμ exon. cDNA from Ramos cell line, which is another Burkitt’s lymphoma derived cell line, and RNA from Jiyoye processed without reverse retrotranscriptase, were used as a positive and negative control respectively. The μGLT expression was analyzed by real-time PCR. The results were normalized to the RPL38 (Ribosomal protein L38) expression, and referred to the expression of control Jiyoye cells (without doxycycline or LMB treatment).

### Chromatin immunoprecipitation

The chromatin immunoprecipitation assays were carried out as previously described^33^. Immunoprecipitated material was analysed by quantitative real time PCR (see primer sequences in Supplementary Table 1). IgG was used as a negative control. Chromatin immunoprecipitation experiments were performed independently at least two times. The antibodies used for these experiments are listed in Supplementary Table 2.

### Immunoprecipitation

293F cells were transfected using polyethylenimine (PEI) with the following plasmids: pCDNA3.1 constructs encoding the FLAG-tagged WT and mutant forms of AID and plasmids encoding the lysine 20 histone 4 methyltransferases, pCDNA4/T0Suv4-20h1.1-HA, pCDNA4/T0Suv4-20h1.2-HA and pCISuv4-20h2-HA^34, 35^. Whole cell extracts were obtained with RIPA buffer (50 mM Tris-HCl pH 7.8, 150 mM NaCl, 0.5% deoxycholic acid, 0.1% SDS, 1% NP40) and treated with benzonase 40U (Sigma-Aldrich) for 4 hours before immunoprecipitation. After this time, the samples were centrifuged for 10 minutes at 7000 *g*. The supernatant was then diluted with BC-100 buffer (10 mM Tris-HCl pH 7.8, 0.5 mM EDTA, 0.1 mM PMSF, 1 mM DTT, 50% glycerol, 100 mM KCl) and overnight incubated at 4 °C with FLAG-agarose (Sigma-Aldrich) for AID immunoprecipitation, or HA-agarose (Sigma-Aldrich) for SUV4-20H immunoprecipitation. The samples were washed twice with BC-100, 0.05% NP-40 and five times with BC-500 (50 mM Tris-HCl pH 7.8, 2.5 mM EDTA, 0.5 mM PMSF, 5 mM DTT, 50% Glycerol, 500 mM KCl), 0.05% NP-40. The immunoprecipitated proteins were eluted using 0.2 M glycine pH 2.3 and the resulting material was analysed by SDS-PAGE and western blot. The antibodies used for these experiments are listed in Supplementary Table 2.

## Electronic supplementary material

Supplementary Information
