# Supplementary material for: Activation-induced cytidine deaminase targets SUV4-20-mediated histone H4K20 trimethylation to class-switch recombination sites
Source: Sci Rep. 2017 Aug 8;7:7594. doi: 10.1038/s41598-017-07380-9 (PMC5548798; doi:10.1038/s41598-017-07380-9)
Supplement: Supplementary file 1 — Supplementary Information [file 41598_2017_7380_MOESM1_ESM.pdf]

Supplementary Information:

Activation-induced cytidine deaminase targets SUV4-20-mediated histone H4K20 trimethylation to class-switch recombination sites

Virginia C. Rodríguez-Cortez[1], Paloma Martínez-Redondo[2,*], Francesc Català-Moll[1,*], Javier Rodríguez-Ubreva[1], Antonio Garcia-Gomez[1], Ganesh Poorani-Subramani[3,4], Laura Ciudad[1], Henar Hernando[1], Arantxa Pérez-García[5], Carlos Company[1], José M. Urquiza[1], Almudena R. Ramiro[5], Javier M. Di Noia[3,4,6], Alejandro Vaquero[2] and Esteban Ballestar[1]

[1] Chromatin and Disease Group, Cancer Epigenetics and Biology Programme (PEBC), Bellvitge Biomedical Research Institute (IDIBELL), 08908 L'Hospitalet de Llobregat, Barcelona, Spain

[2] Chromatin Biology Group, Cancer Epigenetics and Biology Programme (PEBC), Bellvitge Biomedical Research Institute (IDIBELL), 08908 L'Hospitalet de Llobregat, Barcelona, Spain

[3] Institut de Recherches Cliniques de Montréal, Division of Immunity and Viral Infections, Montréal, H2W 1R7 Québec, Canada

[4] Division of Experimental Medicine, Faculty of Medicine, McGill University, Montreal, QC. Canada

[5] B Cell Biology Lab, Centro Nacional de Investigaciones Cardiovasculares Carlos III (CNIC), Madrid, Spain

[6] Université de Montréal, Department of Medicine, Montreal H3T 1J4 Québec, Canada

*Equal contributors

Supplementary Figure Legends

Supplementary Figure 1. Effects of AID binding in DNA methylation. (A) Schematic representation showing the Sμ and Cμ regions of the IGH locus, the bona fide binding and non-binding sites for AID respectively. The scheme show the specific areas analyzed by pyrosequencing (red arrows) and ChIP assays (blue line). (B) Schematic representation of the results obtained from the DNA methylation profiling of the Jiyoye cell line with the Illumina 450K DNA methylation array. Green ovals represent the different comparisons of DNA methylation profiles. In red, it is indicated the number of significantly hypermethylated CpGs.In blue, the number of significantly hypomethylated CpGs. Red and blue numbers next to the arrows represent the hypomethylated or hypermethylated CpGs that coincide among the different comparisons: before (C) and after induction with doxycycline (D), and following inhibition of nuclear export with leptomycin B (L). The combinations are as follows: C, CL, D, DL (C) Band pattering corresponding to DNA methylation analysis of Alu repeats, using AUMA assays. No significant differences were observed in the analyzed conditions neither in Hela nor in Jiyoye cells. The controls of the assay are constituted by DNA from Jiyoye cell line (without retroviral vectors) and the in vitro methylated DNA from the same cell line (IVD), which is the control of DNA methylation. (D) Effects on the DNA methylation status of Jiyoye cells of the expression of AID

Supplementary Figure 2. (A) Primary structure of AID. The upper part of the diagram indicates the four missense mutations related to HIGM2 used in our study. The lower part indicates the two selected nonsense HIGM2 mutations. NLS, nuclear-localisation signal; CDD, cytidine deaminase domain; L, linker region; ALD, APOBEC-like domain; NES, nuclear-export sequence. (B) Western blot image showing the inducible expression of AID WT and the various HIGM2 mutants, before and after treatment with doxycycline (Doxy) 500 ng/ml for 48 hours in Jiyoye cells. (C) Representative confocal images showing the subcellular localisation of C-terminally hemagglutinin (HA)-tagged human AID in inducible Jiyoye cells. When nuclear export was inhibited with leptomycin B (LMB) 50 ng/ml for 2 hours, most of the AID translocates from the cytoplasm to the nucleus. Protein products of missense HIGM2 mutations showed a similar response to AID WT after LMB, while truncated forms of AID lacking NES, were constitutively nuclear. Scale bar: 10 μm.

A

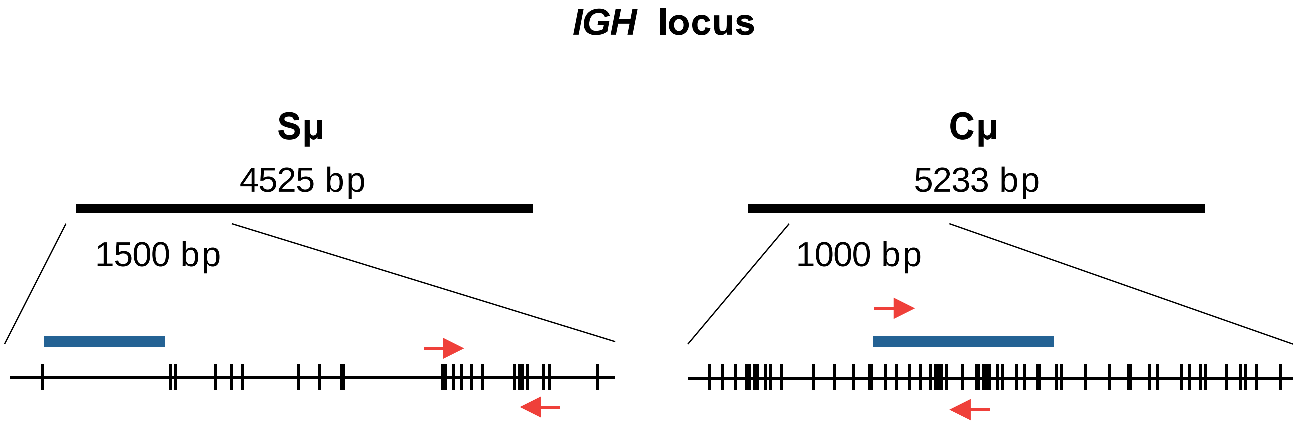
IGH locus
Sμ
4525 bp
1500 bp
Cμ
5233 bp
1000 bp

B

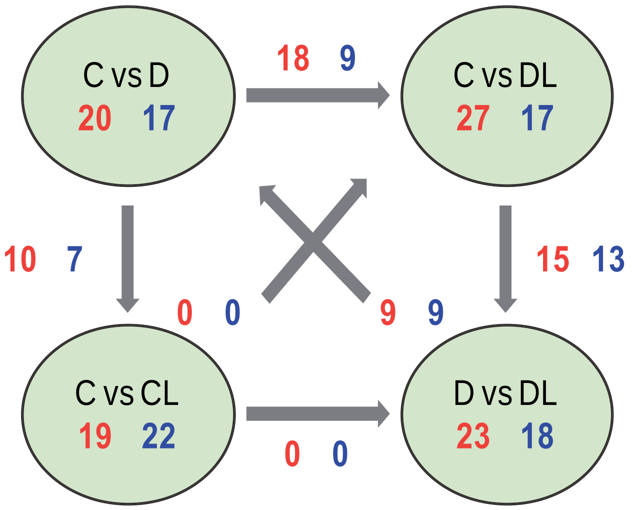
C vs D
20 17
18 9
C vs DL
27 17
10 7
0 0
9 9
15 13
C vs CL
19 22
0 0
D vs DL
23 18

C

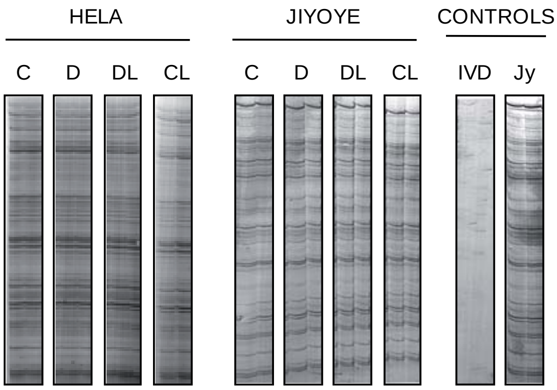
HELA
JIYOYE
CONTROLS
C
D
DL
CL
C
D
DL
CL
IVD
Jy

D

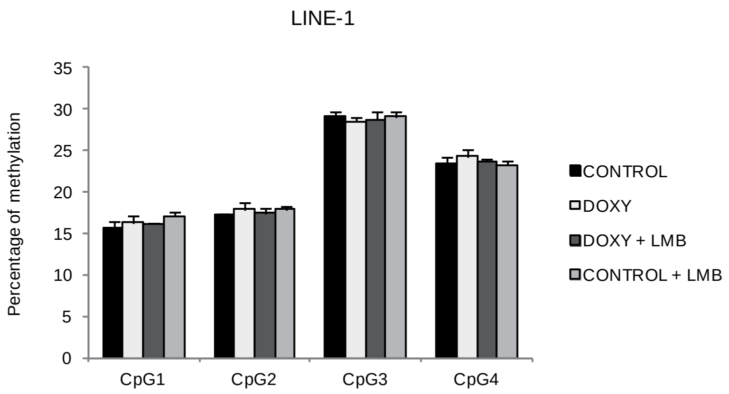
LINE-1
Percentage of methylation
0
5
10
15
20
25
30
35
CpG1
CpG2
CpG3
CpG4
CONTROL
DOXY
DOXY + LMB
CONTROL + LMB

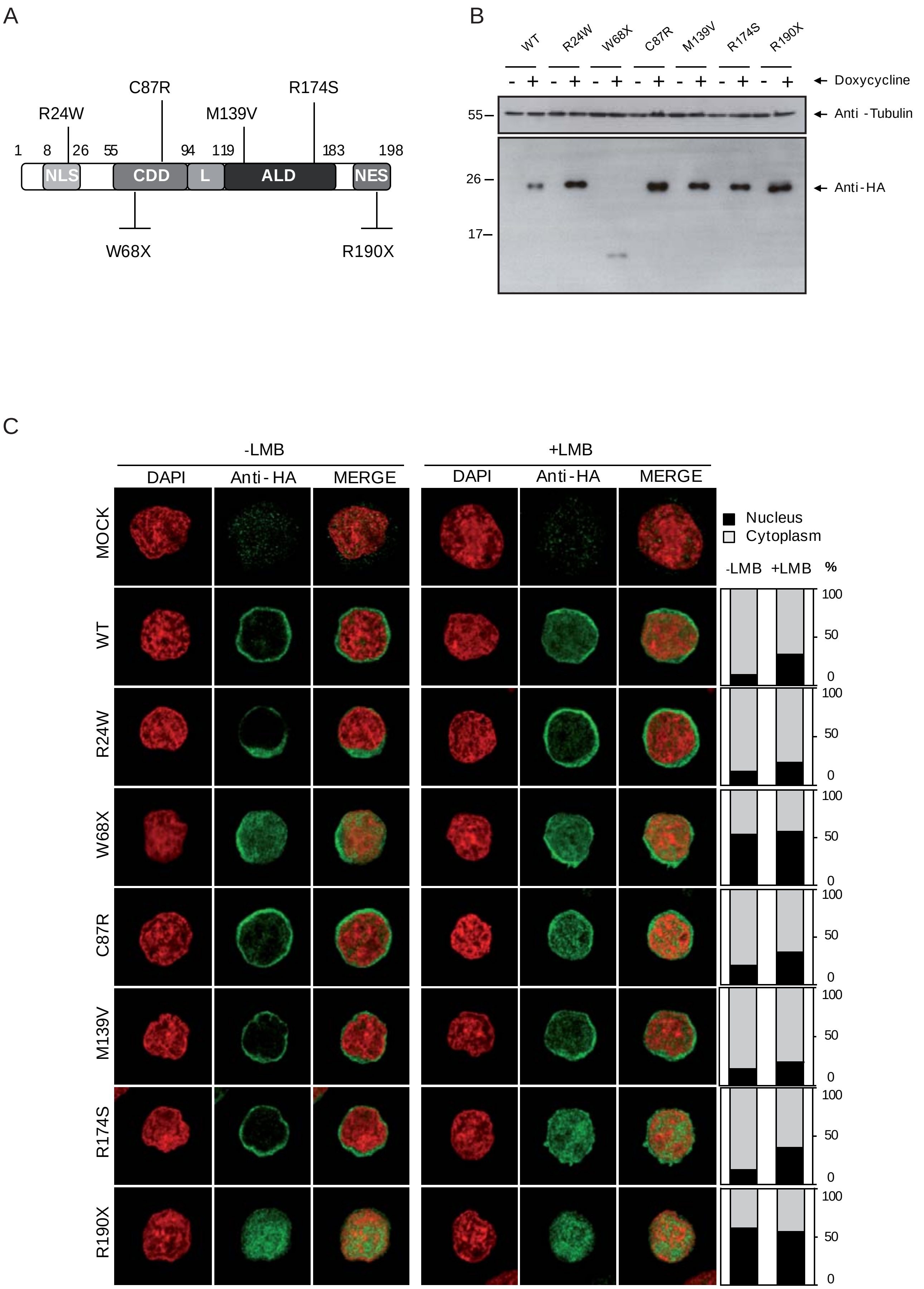
A
R24W
C87R
M139V
R174S
1 8 26 55 94 119 183 198
NLS
CDD
L
ALD
NES
W68X
R190X
B
WT
R24W
W68X
C87R
M139V
R174S
R190X
- + - + - + - + - + - + - +
Doxycycline
55
Anti -Tubulin
26
Anti-HA
17
C
-LMB
+LMB
DAPI
Anti-HA
MERGE
MOCK
WT
R24W
W68X
C87R
M139V
R174S
R190X
Nucleus
Cytoplasm
-LMB +LMB
%
100
50
0

Supplementary Table 1. List of primers

	Forward Primer	Reverse Primer
**Cloning**		
AICDA WT	ATGGATCCAGACACTCTGGACACCACTATG	TAGAATTCCTAAGCGTAATCTGGAACATCGTA
**Site-Directed Mutagenesis**		
R24W (70 C>T)	GCTGGGCTAAGGGTTGGCGTGAGACCTACC	GGTAGGTCTCACGCCAACCCTTAGCCCAGC
W68X (203 G>A)	AAATGTCCGCTGGGCTAAGGGTCGGCGTGA	TAGCACAGGTAGGTCTCACGCCGACCCTTA
C87R (259 T>C)	CCTCCTGGAGCCCCCGCTACGACTGTGCCC	GGGCACAGTCGTAGCGGGGGCTCCAGGAGG
M139V (415 A>G)	TGCAAATAGCCATCGTGACCTTCAAAGATT	AATCTTTGAAGGTCACGATGGCTATTTGCA
R174S (522 A>C)	GTTCGTCTCTCCAGCCAGCTTCGGCGCATC	GATGCGCCGAAGCTGGCTGGAGAGACGAAC
R190X (568 C>T)	AGGTTGATGACTTATGAGACGCATTTCGTA	TACGAAATGCGTCTCATAAGTCATCAACCT
**ChIP**		
**Human:**		
Sμ	TGAGATGGCTTTAGCTGAGACAAG	CAGCTCACCTGGTGCAACTTAG
Cμ	CACGTGGTGTGCAAAGTCCAGCACC	ACGCCAGACCCCACCTGCTT
**Mouse:**		
Em	TCAAGATGGCCGATCAGAACCAGAACACCT	CTTCCCCAAATAGCCTTGCCACATGACCTG
Sm-Upstream	TAGTAAGCGAGGCTCTAAAAAGCAC	ACTCAGAGAAGCCCACCCAT
Sm-Downstream	GGTTGGGAGACCATGAATTG	TTCTTAGCTCAACCCAGTTTATCC
Cm	CTGAACCTGAGGGAGTCAGC	GCCACTGCACACTGATGTCT
Sa-Upstream	GGCTAGAATGGGCTAGAGTGAGTTA	GCCTATTTTGGCCAGTCTACTTAC
Sa-Downstream	CTTGGCTAGGCTACAATGGATTGAGC	GTGCAACTCTATCTAGGTCTGCCCGGT
GAPDH	CACCTTCAGCTTTCCGGCCACTTAC	GGAAGCCCATCACCATCTTCCAGGA
**μGLT expression**		
Iμ	ATGGATCCAGACACTCTGGACACCACTATG	
Iμ		TGCTCTGAGGTATCGAAAAAG
**DNA accesibility assay**		
Sμ	TGAGATGGCTTTAGCTGAGACAAG	CAGCTCACCTGGTGCAACTTAG
Cμ	CACGTGGTGTGCAAAGTCCAGCACC	ACGCCAGACCCCACCTGCTT
D4Z4	CTCAGCGAGGAAGAATACCG	ACCGGGCCTAGACCTAGAAG
c-fos	CGAGCATCTGAGAAGCCAAG	GAAGCCCGAGAACATCATCG
**AUMA**		
Blue adaptor	CCGAATTCGCAAAGCTCTGA	
P-MCA adaptor	^{P}-TCAGAGCTTTGCGAAT	
ALU up 5 primer	ATTCGCAAAGCTCTGAGGGTT	

Supplementary Table 2. List of antibodies

Antibody	Company	Reference	Application
α- hemagglutinin (HA)	Sigma	H6908	WB,IF, ChIP
Monoclonal α- HA - Agarose antibody	Sigma	A2095	Co-IP
α- FLAG	Sigma	F7425	WB
α- FLAG M2 AFFINITY GEL	Sigma	A2220	Co-IP
α- AID	Invitrogen	39-2500	WB
α- histone H3	Abcam	ab1791	WB
α- H3K4me3	Millipore	upstate 07-473	WB, ChIP
α- H3K27me3	Millipore	upstate 07-449	WB, ChIP
α- histone H4	Abcam	ab10158	WB
α- H4K20me3	Millipore	04-079	WB
α- H4K20me3	Millipore	upstate 07-749	ChIP
α- H4K20me3	Abcam	ab9053	ChIP
Goat anti-rabbit conjugated to horseradish peroxidase (HRP)	Amersham	RPN4301	WB
Sheep anti-mouse-HRP	Amersham	RPN4201	WB
Alexa Fluor® 488 Donkey Anti-Rabbit IgG (H+L) Antibody	Life technologies	A-21206	IF
